# Reflections on predictive modeling for infectious diseases

**DOI:** 10.3389/fpubh.2026.1811213

**Published:** 2026-04-13

**Authors:** Daren Zhao

**Affiliations:** 1 Faculty of Social Sciences and Humanities, Mahidol University, Salaya, Nakhon Pathom, Thailand; 2 Quality Control Management Department, Sichuan Provincial Orthopedics Hospital, Chengdu, Sichuan, China

**Keywords:** application, early warning, infectious disease, prediction models, surveillance

## 1 Introduction

The global community has faced substantial threats from infectious diseases in recent decades. As a crucial element of epidemic surveillance systems, infectious disease prediction technology plays an essential role in enabling early warning and facilitating informed decision-making. The convergence of globalization and climate change is exacerbating the threat of emerging infectious diseases, posing unprecedented challenges to global public health (1). Over the past few decades, the global community has experienced a series of emerging infectious disease outbreaks, including SARS, H1N1, COVID-19, and Monkeypox virus, each posing significant threats to public health and inflicting substantial socioeconomic damage across the world (2).

Infectious disease surveillance and early warning serve as a critical tool for public health response, providing essential evidence for outbreak prevention and control and delivering vital strategic reference for health administrative departments (3). Simultaneously, this function establishes it as a crucial epidemiological tool for continuous population public health monitoring (4). Therefore, during an infectious disease outbreak, scientific early warning of incidence provides critical support for predicting epidemic trends and facilitates proactive allocation of medical resources (5). As a result, the preparedness and response capabilities of healthcare systems are significantly enhanced.

**Figure 1:**
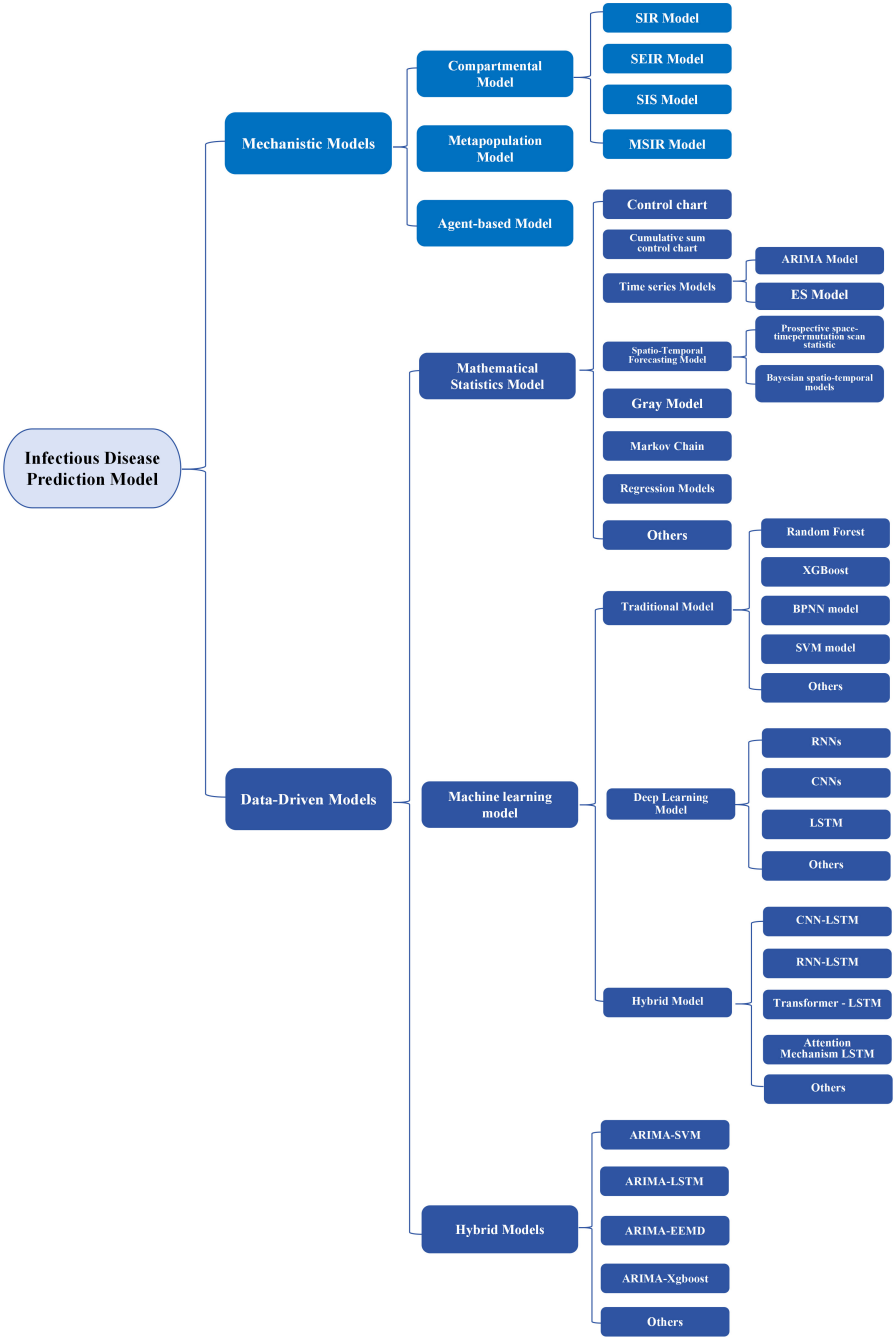
Classification chart of infectious disease prediction models.

A systematic review of relevant literature indicates that a suite of validated predictive models can be employed for forecasting infectious disease trends. In contrast to earlier classification schemes (3, 6), our study categorizes infectious disease prediction models into two primary types: mechanistic and data-driven. This classification is based on their core theoretical principles, model construction methodologies, and empirical evidence of effectiveness, with further subdivision according to specific modeling procedures and practical usage scenarios. A comprehensive overview of the model classification is presented in Figure 1.

As illustrated in Figure 1, both mechanistic and data-driven models encompass a variety of sub-models, from which new model types and a growing number of variants continue to emerge. This trend demonstrates that infectious disease prediction modeling techniques have been continuously advancing worldwide in recent decades. However, irrespective of how predictive models are categorized, the central objective remains using these models to forecast the future transmission dynamics of infectious diseases. Therefore, the accuracy of predictions, as well as their robustness, serves as a critical criterion for evaluating model performance.

In real-world settings, the predictive accuracy of models is influenced by multiple factors, including inherent model limitations and socioeconomic variables (7). This often results in a discernible “predictive deviation” between theoretical projections and the actual incidence of infectious diseases. Indeed, addressing this “predictive deviation” poses a fundamental challenge in the practical application of modeling frameworks within contemporary infectious disease epidemiology. However, this focus on addressing “predictive deviation” has largely centered on final model performance, leaving the robustness of learning processes and the mechanisms underlying generalizability comparatively under-examined.

## 2 Challenges

The continual development of novel predictive models drives the advancement of forecasting techniques aimed at reducing the gap between theory and practice and mitigating “predictive deviation.” However, their practical utility remains constrained by fundamental challenges pertaining to data availability and quality, methodological appropriateness and validity, and ethical implications. Therefore, the process of implementing such models in real-world infectious disease forecasting is fraught with numerous challenges. These challenges manifest primarily in three interconnected dimensions.

First, there are significant obstacles concerning data availability and quality. In many cases, during the early stages of an infectious disease outbreak, case data may be incomplete and of suboptimal quality (8). As a result, the models developed to simulate the actual occurrence and transmission dynamics of the disease are subject to limitations. As a consequence of disparities in population coverage, modes of data acquisition, data quality, and the limited utility of available data restricts forecasting performance, leading to diminished predictive accuracy in modeling (9, 10).

Second, there arise considerable methodological challenges relating to appropriateness, validity, and robustness. Given that each class of predictive model possesses inherent strengths and limitations, the selection of the most appropriate framework is a prerequisite for achieving reliable predictive outcomes (11). Specifically, model selection is critically dependent upon the sources, characteristics, and distribution of the data (12). The complex mechanistic nature inherent in infectious disease incidence data means that the uncritical application of novel modeling approaches for outbreak prediction may result in substantially compromised model interpretability. Model validity and robustness represent a dual aspect of predictive methodology: they are both a fundamental objective and its greatest methodological challenge. Frequent overfitting and poor generalizability often prevent predictive models from being used effectively in real-world disease surveillance (13). A concrete example is data leakage during data preprocessing, which occurs when random record-level splitting of patient data without patient-level clustering, a common practice among machine learning practitioners lacking clinical domain expertise, inadvertently assigns multiple data points from the same patient to different dataset partitions (14). Such data leakage enables models to achieve high accuracy on held-out test sets by memorizing patient-specific patterns rather than learning generalizable disease dynamics, leading to substantially overoptimistic performance estimates (15). These errors highlight the need for integrating clinical expertise into model development to ensure patient-level clustering during dataset partitioning, and for educational reforms that prepare future clinicians to oversee AI development and uphold critical methodological safeguards (16).

Finally, privacy and justice remain persistent and critical challenges in infectious disease forecasting. The rapid evolution of predictive modeling techniques, particularly the iterative advancement of machine learning-based prediction models, has significantly accelerated progress in infectious disease forecasting. However, this very progress raises critical concerns regarding privacy, equity, and other ethical implications. The deployment of artificial intelligence typically necessitates access to extensive sensitive health data, which raises important concerns regarding patient confidentiality (17). For instance, machine learning predictive models often require large-scale datasets for training, frequently involving the aggregation of detailed personal health information—including clinical, diagnostic, laboratory, and health service records (18). Furthermore, since the predictions generated by models may directly or indirectly influence public health interventions, they can introduce ethical challenges pertaining to autonomy and justice (19).

## 3 Summary and outlook

First, data quality control must be strengthened. Data quality is one of the most important drivers of model performance (20). High-quality data require not only standardized real-time reporting but also rigorous quality control for confirmed cases. This is particularly important during the early stages of an outbreak, when clinical diagnoses are often unclear and underreporting or misreporting may occur. Enhanced quality assurance is essential to ensure the reliability and authenticity of surveillance data used for forecasting.

Second, interdisciplinary integration is crucial in predictive methodology. Infectious disease forecasting is a complex endeavor that involves multiple disciplines, including epidemiology, climate science, demography, data science, and technology (21). It is therefore essential to establish cross-disciplinary research teams and promote integrated approaches to advance the field of epidemic prediction. A representative example is the global COVID-19 prediction system developed by Huang et al. (22), which exemplifies such integration by combining atmospheric science (statistical-dynamical climate models), public health (SIR epidemiological modeling), environmental science (NO_2_ concentration as a proxy for lockdown intensity), and political science (analysis of government interventions). This operational system has informed real-time public health decision-making throughout the pandemic (22). Furthermore, ensemble forecasting integrates the strengths of multiple models to generate more robust prediction intervals (23). Methodologically, greater emphasis should be placed on out-of-sample validation and stress-testing to enhance model generalizability. Meanwhile, adherence to reporting guidelines such as EPIFORGE 2020 is recommended to improve transparency and standardize communication in epidemic forecasting research (24).

Third, a robust framework for model application should be established and continuously refined. As an ongoing process that can be implemented in the immediate future, we call for enhanced ethical awareness within the infectious disease modeling community. The framework should be developed and updated through multi-stakeholder collaboration to establish ethical guidelines for infectious disease prediction models based on principles including autonomy, justice, and beneficence, thereby ensuring prediction fairness and strengthening data privacy protection (19). Furthermore, model developers should integrate ethical impact assessments during the initial design phase rather than relying solely on *post-hoc* evaluations (19).

Finally, a focus on aggregate metrics such as final test accuracy is insufficient for fully evaluating model generalization. Models that perform well on held-out test sets may still exhibit unhealthy learning dynamics during training, including erratic loss trajectories, persistent divergence between training and validation loss, or failure to converge. Therefore, model validation should incorporate training dynamics indicators, such as learning curves and convergence behavior, to determine whether the model has learned generalizable patterns or merely memorized noise in the training data (25). For instance, deep neural networks can fit completely randomized labels with zero training error, yet perform no better than random chance on test data (26). This finding suggests that strong training set performance may stem from memorization rather than the acquisition of generalizable patterns. Consequently, evaluating models solely on test set accuracy carries inherent limitations, as models with strong performance may merely have memorized noise in the training data (26). Medical AI models are prone to “shortcut learning,” where predictions rely on spurious correlations in the training data (27). Such spurious correlations may stem from demographic attributes or medical device artifacts (27). As these biases may shift between the training set and external deployment environments, models that depend on shortcuts face a risk of generalization failure when deployed (27). Therefore, this issue warrants serious attention from researchers: In infectious disease forecasting, neglecting learning dynamics can cause significant discrepancies between a model's apparent success in validation and its real-world performance. Such discrepancies undermine predictive reliability and risk biasing public health interventions. Future research should therefore integrate learning dynamics analysis into model validation to enhance robustness and applicability (25, 28).
